# Tyrosinase Inhibitory Effects and Antioxidative Activities of Saponins from *Xanthoceras Sorbifolia* Nutshell

**DOI:** 10.1371/journal.pone.0070090

**Published:** 2013-08-21

**Authors:** Hongmei Zhang, Quancheng Zhou

**Affiliations:** School of Agricultural Engineering and Food Science, Shandong University of Technology, Zibo, Shandong Province, China; Rochester Institute of Technology, United States of America

## Abstract

Certain saponins are bioactive compounds with anticancer, antivirus and antioxidant activities. This paper discussed inhibitory effects of saponins from *Xanthoceras Sorbifolia* on tyrosinase, through the research of the rate of tyrosinase catalyzed L-DOPA oxidation. The inhibition rate of tyrosinase activity presented non-linear changes with the saponins concentration. The rate reached 52.0% when the saponins concentration was 0.96 mg/ml. Antioxidant activities of saponins from *Xanthoceras Sorbifolia* were evaluated by using hydroxyl and superoxide radical scavenging assays. The hydroxyl radical scavenging effects of the saponins were 15.5–68.7%, respectively at the concentration of 0.18–2.52 mg/ml. The superoxide radical scavenging activity reduced from 96.6% to 7.05% with the time increasing at the concentration of 1.44 mg/ml. All the above antioxidant evaluation indicated that saponins from *Xanthoceras Sorbifolia* exhibited good antioxidant activity in a concentration- dependent manner.

## Introduction

Tyrosinase (EC 1.14.18.1) is a copper-containing oxidase with diverse physiological roles related to melanin production [1]. It catalyses the two reactions by hydroxylation of monophenols and by oxidation of o-diphenol into the corresponding o-quinone, which interact with other molecules to non-enzymatical polymerise to form brown or black pigments [2]. The browning in vegetables and fruits generally results in a less attractive appearance and a loss in nutritional quality [3]. Moreover, abundant generation and excessive activation of tyrosinase can cause various dermatological disorders such as melasma, freckles, age spots, and sites of actinic damage [4]. In addition, tyrosinase is also responsible for melanin distribution in animals [5]. Reactive quinone intermediates in the melanin biosynthetic pathway exhibit antibiotic properties. Consequently, searching tyrosinase inhibitors will be important for inhibition of enzymatic browning in plants, prevention of agricultural insect pest and pigmentation disorders. Furthermore, they can also be used in medicinal and cosmetic whitening agents [6].

Antioxidants are defined as substances that can delay or prevent oxidative cellular oxidisable substrates. They work via two ways: scavenging reactive oxygen species (ROS) or inhibiting the generation of ROS. At present, synthetic antioxidants are widely used for Whitening cosmetics and industrial processing to preserve food quality. However, some reports have been suspected of the side effects and stability of some common ones. For example, hydroquinone has been associated with mutagenicity and the incidence of ochronosis in African countries [7]. Thus, there has been increasing interest in exploring natural, safe and effective alternative sources, since they can protect the human body from free radicals and retard the progress of many chronic diseases [8]. Published data indicate that some plant saponins have antioxidant activities and can be explored as novel potential antioxidants [9], [10]. In addition, saponins extracted from *Xanthoceras Sorbifolia* nutshell, have also shown antioxidant properties as shown by their free radical scavenging abilities.

The importance of free radicals and ROS in the production of energy fuel biological processes has attracted increasing attentions over the past years. But it is also essential in cellular damage and the ageing process. ROS, such as hydroxyl radical, superoxide anion radical and other free radicals are by-products of biological metabolism. They are associated with the destruction and deterioration of food quality through lipid, proteins, and nucleic acids oxidation during storage and induce to ageing and various diseases, such as cancer, diabetes mellitus, cardiovascular diseases, skin disorders and neurodegenerative diseases [11].

*Xanthoceras Sorbifolia* (*Xanthoceras Sorbifolia* Bunge, family Sapindaceae) is an indigenous shrub distributed in northeast and north of China with a lifespan of more than 200 years. And as an important energy plant for bio-diesel [12] with more than 60% oil in the kernel [13], it can be utilized to produce biodiesel as a substitute for petrol to relieve the serious situation of energy consumption [14]. *Xanthoceras Sorbifolia* has been directly eaten in China for a long time as traditional folk medicine to treat nocturnal enuresis and dysentery. It also has been used in traditional medicine for curing arterial sclerosis, hyperlipemia, hyperpiesia, chronic hepatitis [15], rheumatism. In addition, studies found that it could significantly ameliorate the impairment of learning and memory in animal models [16] and inhibit human cancer cell lines [17]. According to previous studies, *Xanthoceras Sorbifolia* contains a variety of bioactive constituents, such as triterpenoid saponins [18], flavonoids and sterols [19]. Triterpenoid saponins, the main constituents of *Xanthoceras Sorbifolia*, had been previously obtained from this plant and proved to have many activities.

Saponins are natural surfactants, found in many plants which are glycosides with a distinctive foaming characteristic. They consist of a polycyclic aglycone that is either a steroid or triterpenoid attached at C3 with a sugar side chain. Triterpene saponins, a large class of plant secondary metabolite can exhibit cytotoxic, haemolytic, molluscicidal, antifungal, antibacterial, antiparasitic, and antiviral activity [20]. Nowadays most attention had been paid to the oil extraction from kernels [21], [22]. Large amounts of shells are discarded as useless residue after oil extraction. Given the waste of bioresource, it is essential to obtain the saponins from the useless residue parts of the plant.

The content of this paper was to test the antioxidant capacity and explore the scavenging effect on radicals of saponins from the defatted residue of *Xanthoceras Sorbifolia* kernel for seeking new biological functional principle used in food and pharmaceutical industry. The aim of our work was to make full use of *Xanthoceras Sorbifolia* and to avoid the waste of this resource after extraction process in oil industrial.

## Materials and Methods

### Experimental Materials

Tyrosinase with 30 U/mg of its enzymatic activity was purchased from Sigma Chemical Co. (St.Louis, MO, USA), L-DOPA, and *Xanthoceras Sorbifolia* was purchased from Shaanxi Jindao seed Industry Company. All other used chemicals were of analytical grade.

### Preparation of sample solutions of saponins

A standard procedure was followed for the isolation of the saponins. The nutshell of *Xanthoceras Sorbifolia* dried and powdered. The powder (200 g) was defatted with 5 times petroleum ether(60∼90°C) at 70°C for 4 h by reflux condensing, then digested in 7 times 70% ethanol at 70°C for 5 h and later filtered. The residue was further extracted with 1400 ml of 70% ethanol for 5 h twice. The combined ethanol extract were concentrated in a rotary evaporator under reduced pressure at 60°C. Then the concentrated solution was dissolved in distilled water (eight times the volume of ethanol extract), followed by centrifugation at 3500 rpm for 15 min. The supernatant liquid was extracted by isometric ethyl acetate for 3 times. Then the aqueous phase was extracted by n-butyl saturated with water for 3 times and finally concentrated in a rotary evaporator.

### Dynamic absorption and desorption tests

Dynamic adsorption and desorption tests were carried out in a glass columns (20 mm×400 mm) wet-packed with 45 g (dry weight) of the selected AB-8 resin. The bed volume (BV) of resin was 70 ml. All dynamic adsorption and desorption experiments were performed at room temperature. Sample solution flowed through the glass column at the flow rate of 1 ml/min and the concentrations of saponins in the effluent liquid were analyzed by UV analysis. After reaching adsorptive saturation, the column was eluted by 75% ethanol (V/V) with a flow rate of 1 ml/min. The concentrations of saponins in the desorption solution were monitored. The combined ethanol liquid were collected and concentrated in a rotary evaporator and lyophilized in a freeze dryer.

### Tyrosinase assay

Inhibitory effects of saponins from *Xanthoceras Sorbifolia* on tyrosinase were determined by the method of Duan, Liu, and Xie [23] with some modifications. In this investigation, L-DOPA was used as a substrate for the tyrosinase activity assay. The reaction media (3 ml) for activity assay contained 0.4 ml (1.0 mg/ml) L-DOPA in Na_2_HPO_4_ – NaH_2_PO_4_ buffer (pH 6.8). The reaction was carried out at a constant temperature of 30°C for 10 min. Then 0.2 ml (250 U/ml) tyrosinase was added into the well. The saponins isolated from *Xanthoceras Sorbifolia* nutshell were accurately weighed, and dissolved by distilled water at different concentration. It was used as a positive control, and distilled water was added instead of the test sample as a blank. The increase in absorbance from 0 to 7 min was measured at 475 nm. Relative enzymatic activity and the inhibitory activity were calculated using the following two equations:
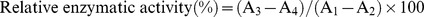


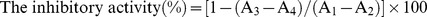
Where *A_1_* is the absorbance which test has L-DOPA without sample, *A_2_* is the absorbance which test has no L-DOPA or sample, *A_3_* is the absorbance which test has both L-DOPA and sample, *A_4_* is the absorbance which test has sample without L-DOPA.

The extent of inhibition by the addition of the sample was expressed as the inhibition ratio. The inhibition type was assayed by the Lineweaver– Burk plot.

### Hydroxyl radicals Assay

In vitro antioxidant activity was evaluated using the hydroxyl radical- system generated by the Fenton reaction [24] with a minor modification. Briefly, samples were dissolved in distilled water at different concentration. The reaction mixture(10 ml) contained 0.75 ml of o-phenanthroline (7.5 mM), 2.5 ml of Na_2_HPO_4_ –NaH_2_PO_4_ buffer (pH 7.4), 0.75 ml of FeSO_4_ (7.5 mM), 1.0 ml of H_2_O_2_(0.10%) and 1 ml samples of varying concentrations in the distilled water. After incubation in the 25°C water bath for 1 h, the absorbance of the mixture was measured at 510 nm. Ascorbic acid was used as the positive control. The hydroxyl radical-scavenging ability was calculated as follows:
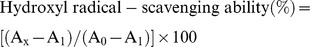
Where A_0_ is the absorbance without samples and H_2_O_2_, A_1_ is the absorbance without samples and A_x_ is the absorbance in the presence of the samples of saponins from *Xanthoceras Sorbifolia*.

### Superoxide radical assay

A method as described by Chen et al. [25] was used to measure superoxide anion scavenging ability of saponins from *Xanthoceras Sorbifolia* with a minor modification. Different concentration of sample was prepared in distilled water. The reaction mixture contained 1 ml of sample solution 3.2 ml distilled water and 4.5 ml Tris-Hcl buffer (pH 8.2). After mixing thoroughly and incubating in the 25°C water bath for 20 min, 0.3 ml of pyrogallol (3.0 mM) was added and measured the absorbance at 320 nm immediately. Scorbic acid was used as positive control. The scavenging activity of superoxide radicals (%) was calculated according to the following equation:
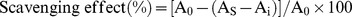
Where A_0_ was the absorbance of the control (distilled water instead of sample); A_S_ was the absorbance of the test sample mixed with reaction solution; A_i_ was the absorbance of the test sample mixed with reaction solution in which HCl (1.0 mM) instead of pyrogallol (3.0 mM).

## Results and Discussion

### Effect of the saponins isolated from Xanthoceras Sorbifolia nutshell on the tyrosinase activity

**Figure 1:**
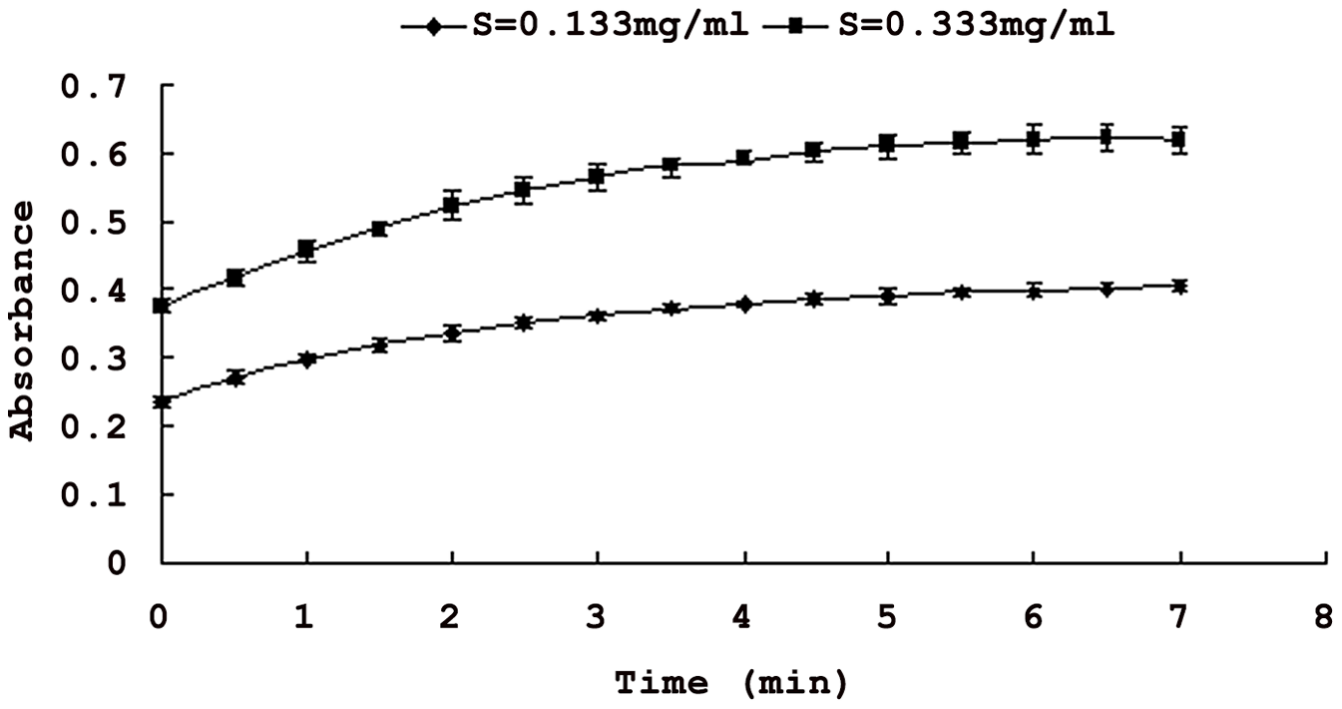
Progress of oxidation of L-DOPA catalyzed by tyrosinase vs. time. **Each value is expressed as mean ± standard deviation (n = 3).**

The progress curve of the oxidation reaction of L-DOPA by tyrosinase was a line (Figure 1), which indicated that the formation of product was in proportion to the reaction time. Absorbance of the reaction system increased over time. But the value of the slope of the line decreased which indicated the tyrosinase activity.

**Figure 2:**
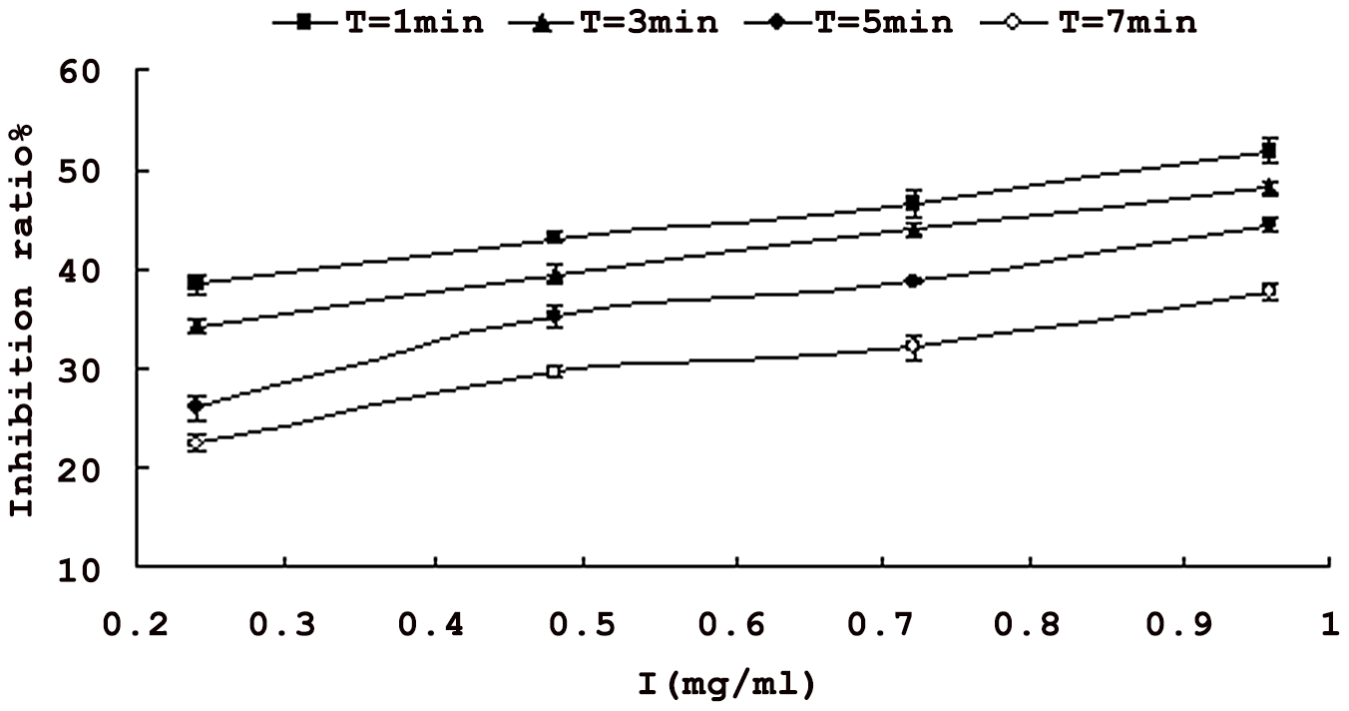
Inhibitory effect of the saponins from *Xanthoceras Sorbifoli* nutshell on activity of tyrosinase. **Each value is expressed as mean ± standard deviation (n = 3).**

Using the saponins isolated from *Xanthoceras Sorbifolia* nutshell as inhibitor, the inhibitory effect of concentration on the tyrosinase activitywas assayed. The inhibitory of tyrosinase activity increased with increasing inhibitor concentrations. When the concentration of inhibitor reached 0.72 mg/ml, enzyme activity was inhibited by 52% (Figure 2). Therefore, the saponins can inhibit the activity of tyrosinase.

**Figure 3:**
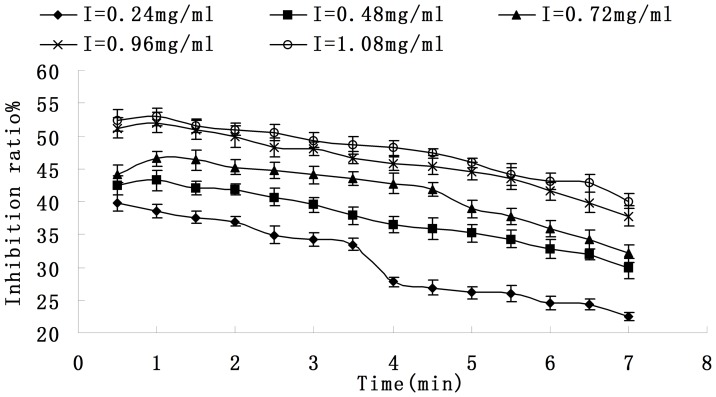
Relationship between reaction time and inhibitory effect on activity of tyrosinase. **Each value is expressed as mean ± standard deviation (n = 3).**

Figure 3 shows the relationship between reaction time and Inhibitory effect on activity of tyrosinase. The system had the highest inhibition ratio at the beginning but slowed down with the time increasing. In the presence of the saponins (1.08 mg/ml), the inhibition ratio was 52.9% on incipient stage. Then it decreased to 40.1%.

### Inhibitory type of the saponins isolated from Xanthoceras Sorbifolia nutshell on the tyrosinase

**Figure 4:**
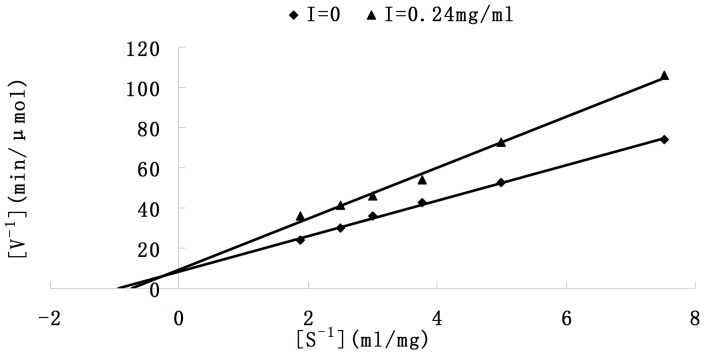
Lineweaver-Burk curve of enzyme reaction rate and the concentration of subtract.

The kinetic behaviour of tyrosinase was studied during the oxidation of L-DOPA. Under the conditions applied in the present study, the inhibitory types of the saponins on tyrosinase activity were determined from Lineweaver–Burk double reciprocal plots. As shown in Figure 4, in the presence of different concentrations (0 and 0.24 mg/ml) of *Xanthoceras Sorbifolia* saponins, the plots of 1/V versus 1/[S] give two straight lines with different slopes. Two lines intersect with each other in the beta quadrant, which indicated that *Xanthoceras Sorbifolia* saponins was not a pure competitive or a pure uncompetitive inhibitor of tyrosinase. According to the line, the value of Km without inhibitor was 1.05 mg/ml. The saponins increased the value of K_m_, but slowed down the oxidation rate, indicated by lower V_max_ values. It exhibited a mixed inhibitory effect towards tyrosinase activity.

The inhibition of the saponins on the tyrosinase was first studied. But many studies have tested the activity of tyrosinase by the oxidation of L-DOPA. However, kinetic values, such as Km and Vmax varied in the different studies. In general, Km obtained in this study was bigger than other reports [26], [27]. The reason for the inconsistency of the kinetic values among different studies is that the oxidation activity of tyrosinase is affected by pH of reaction buffer, enzyme, as well as the types of hydrogen donors, such as DOPA. Moreover, the different substrates used in different works may result in different conclusions.

### Scavenging activity of hydroxyl radical

**Figure 5:**
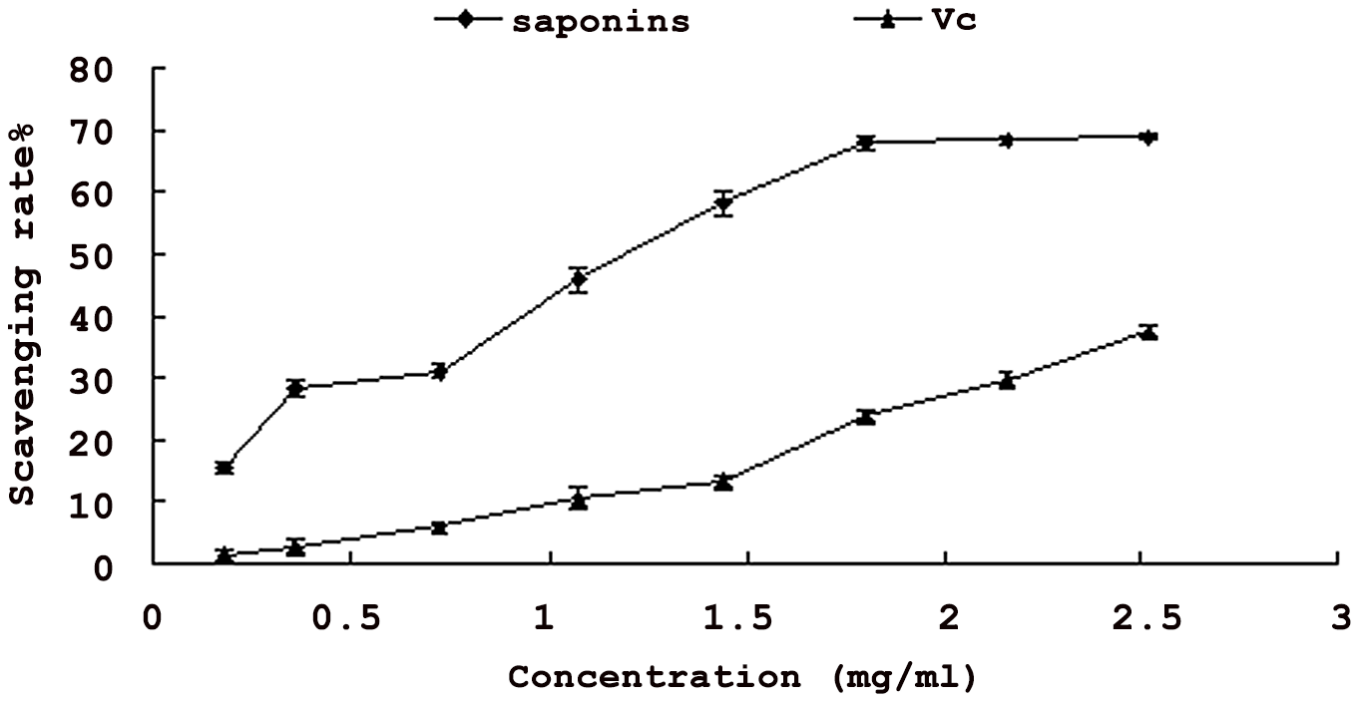
Hydroxyl radical-scavenging activity of the saponins and Vc. **Each value is expressed as mean ± standard deviation (n = 3).**

Generation of reactive oxygen species (ROS) beyond the body's antioxidant capacity gives rise to oxidative stress. Much of the oxidative damage to biomolecules can be induced by hydroxyl radical, the most reactive one among ROS species [28]. The removal of hydroxyl radical is therefore probably one of the most effective defences of a living body against various diseases. As illustrated in Figure 5, saponins from *Xanthoceras Sorbifolia* nutshell had higher scavenging effect than vitamin C. Their scavenging effects increased with increasing concentration. The scavenging effects of the saponins were 15.5–68.7%, respectively at the concentration of 0.18–2.52 mg/ml, and that of Vc was about 1.13–37.6%. These results proved that saponins from nutshell of *Xanthoceras Sorbifolia* had a significant effect on scavenging hydroxyl radicals, and saponins were more pronounced than Vc. Because of the complex mechanism of antioxidant activity, one test is normally not enough to evaluate precisely the antioxidant activity of the potential antioxidant. So various oxidative stress mediated injury models for the saponins should deserve more research in the future.

### Scavenging activity of superoxide radical

**Figure 6:**
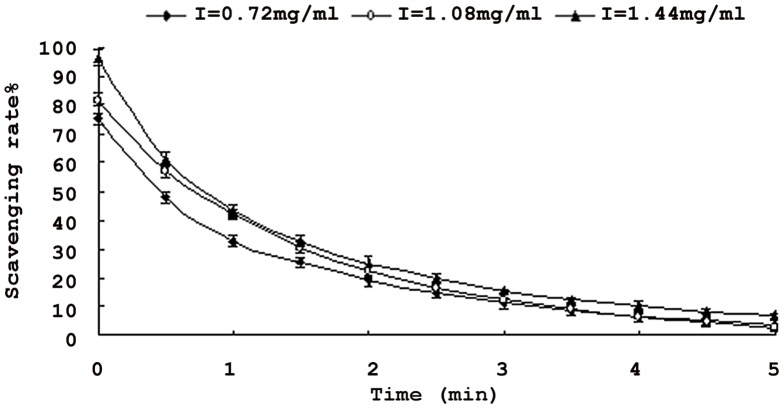
Scavenging effects of saponins from *Xanthoceras Sorbifolia* nutshell on super oxide radicals. **Each value is expressed as mean ± standard deviation (n = 3).**

Superoxide radical is known to be very harmful to cellular components as a precursor of more reactive oxidative species, such as single oxygen and hydroxyl radicals, which is considered to play an important role in the peroxidation of lipids [29]. Thus, it is important to remove superoxide radicals. Figure 6 showed that superoxide radical scavenging activities decreased with the time increasing. When the saponins content was at 0.72 mg/ml, scavenging activity reduced from 75.3% to 4.07%. When at 1.44 mg/ml, scavenging activity reduced from 96.6% to 7.05%. It clearly indicates that the saponins exhibited the highest superoxide radical-scavenging effect at the beginning. Meanwhile, superoxide radical-scavenging activities increased with increasing sample concentrations. Therefore saponins from nutshell of *Xanthoceras Sorbifolia* might exhibit a scavenging of superoxide radicals.

### Conclusion

*Xanthoceras Sorbifolia* as an energy plant has been grown in China for a long time. To our knowledge, this is the first report demonstrating that the saponins from *Xanthoceras Sorbifolia* nutshell were a good tyrosinase inhibitor. Moreover, it had antioxidant activity, as seen in hydroxyl radical-scavenging activity, superoxide radical- scavenging activity assay. On the basis of the previously mentioned results, it was concluded that saponins from *Xanthoceras Sorbifolia* nutshell had higher scavenging activity of hydroxyl radical than that of Vc.

Overall, we can rationally assume that saponins from *Xanthoceras Sorbifolia* nutshell could be useful in the treatment of some skin hyperpigmentation disorders and inhibition of the browning response of fruits and vegetables in the future. It also had good antioxidant properties and can be developed as a novel potential antioxidant for the treatment and prevention of some diseases relating to ROS. Thus, *Xanthoceras Sorbifolia* nutshell should be explored and investigated further in view of its potential.
